# Sonothrombolysis for Ischemic Stroke

**DOI:** 10.3390/jcdd11030075

**Published:** 2024-02-22

**Authors:** Narayanaswamy Venketasubramanian, Leonard L. L. Yeo, Benjamin Tan, Bernard P. L. Chan

**Affiliations:** 1 Raffles Neuroscience Centre, Raffles Hospital Singapore, Singapore 188770, Singapore; 2 Division of Neurology, National University Hospital, Singapore and Yong Loo Lin School of Medicine, National University of Singapore, Singapore 119074, Singapore; leonardyeoll@gmail.com (L.L.L.Y.); benjamin_yq_tan@nuhs.edu.sg (B.T.); bernard_chan@nuhs.edu.sg (B.P.L.C.)

**Keywords:** ischemic stroke, sonothrombolysis, ultrasound, thrombolysis

## Abstract

Stroke is a major cause of death and disability globally, with ischemic stroke being the predominant mechanism. While spontaneous recanalization may occur, significant neuronal injury would have occurred in the interim. Intravenous thrombolysis administered within the first 4.5 h after stroke onset and endovascular thrombectomy within 24 h in patients with a salvageable penumbra improves functional independence. Ultrasound has been shown in both in vivo and in vitro models to enhance clot lysis, even more-so in the presence of thrombolytic agents. The use of transcranial Doppler and transcranial color-coded Doppler ultrasound in acute IS has been reported in case series, case-controlled studies, and clinical trials. While ultrasound at a frequency of 300 kHz increases the risk of intracranial hemorrhage, the 2 MHz range ultrasound aids thrombolysis and improves recanalization without significantly increasing the risk of symptomatic intracranial hemorrhage. Despite this, functional independence was not increased in clinical trials, nor was a benefit shown with the adjunctive use of microbubbles or microspheres. Nonetheless, newer technologies such as endovascular ultrasound, endovascular delivery of microbubbles, and thrombolytic-filled microbubbles await clinical trials. More evidence is needed before sonothrombolysis can be routinely used in the hyperacute management of ischemic stroke.

## 1. Introduction

Stroke is a major cause of health burden globally. Based on the Global Burden of Disease Study, in 2019, stroke incidence was 12.2 million cases (95% UI 11.0–13.6), with a prevalence of 101 million cases (95% UI 93.2–111) and a mortality of 6.55 million cases (95% UI 6.00–7.02), with 143 million (95% UI 133–153) disability-adjusted life years (DALYs) lost due to stroke [1]. Among incident cases, the most common cause of stroke was arterial occlusion causing ischemic stroke (IS) at 62.4% (7.63 million [95% UI 6.57–8.96]), with 27.9% (3.41 million [95% UI 2.97–3.91]) due to intracerebral hemorrhage (ICH), and 9.7% (1.18 million [95% UI 1.01–1.39]) due to subarachnoid hemorrhage (SAH).

It is estimated that in patients with IS, due to acute large arterial occlusion, in each hour, 120 million neurons, 830 billion synapses, and 714 km of myelinated fibers are lost; in each minute, the respective numbers are 1.9 million, 14 billion, and 12 km [2]. This has bolstered the concept that ‘time is brain’ and the need to begin treatment as soon as possible. Thus, among the key approaches in the management of acute IS is emergent and rapid revascularization of the occluded artery to re-establish adequate perfusion to ischemic tissue so as to rescue as much brain tissue as possible and limit further damage caused by ongoing ischemia [3].

## 2. Recanalization after Ischemic Stroke

In a systemic review of 10 angiographic studies among patients with IS (*n* = 1130), the authors found that spontaneous recanalization (SR) occurred in approximately 17% of patients in the first 6 to 8 h after stroke onset, with a lack of occlusion seen in 28% to 50% of patients by four days [4]. This shows that some SR occurs naturally after large vessel occlusion, but the proportion is small in the early hours after IS onset, and still poor even after four days, which, in the absence of adequate collateral flow, can lead to brain tissue continuing to be damaged from inadequate perfusion and ongoing ischemia. It would be difficult to obtain similar data on small vessel occlusion due to their tiny size and difficulty in their visualization on cerebral angiography, but as these are usually end-arteries with little collateral flow, the ischemic injury, although to a smaller volume of brain tissue, is likely to be more severe within that territory.

As a corollary, in a study of adults, 38 with IS compared to 17 healthy controls, central motor conduction times (CMCTs) at day 14 post-stroke were improved if spontaneous recanalization occurred within 24 hours compared to after 24 h (87% vs. 62% of patients, *p* = 0.005); CMCT improved in only 17% of those with no recanalization [5]. Thus, early recanalization is crucial. Delayed recanalization, even if it occurs, may not only be unhelpful in aiding stroke recovery due to irrecoverable tissue damage from prolonged inadequate perfusion, but it also carries the serious risk of reperfusion vasogenic oedema and hemorrhagic transformation, which may be deleterious to the already fragile ischemic tissue [6,7].

The process of SR involves the natural lysis of the fibrin network that holds the clot together, allowing blood to flow past the site of occlusion [8]. SR is achieved by plasmin-dependent and plasmin-independent pathways. Of the plasmin-dependent pathways, vascular endothelial cells produce activators of fibrinolysis, such as the tissue-type plasminogen activator (tPA) and the urokinase-type plasminogen activator (uPA). These activators convert circulating plasminogen to plasmin, which then breaks down fibrin and lyses the clot. Plasmin-independent pathways involve the accumulation of polymorphonuclear leucocytes at the site of the clot that then activate plasmin-dependent pathways as well as release serine proteases, including proteinase 3, cathepsin G, and elastase, that break up the clot. Monocytes infiltrating into the clot also release plasminogen activators that then cause clot lysis.

Various thrombolytic agents have been developed that are able to enhance the intrinsic clot lysis by their ability to convert circulating plasminogen to plasmin, which then lyses the clot [9]. Such agents include alteplase, duteplase, desmoteplase, reteplase, staphylokinase, streptokinase, pro-urokinase, urokinase, tenecteplase, and anistreplase (APSAC), to name a few. In a meta-analysis of 27 randomized trials (*n* = 10,187), thrombolytic therapy, mostly given up to six hours after the onset of IS, significantly reduced the odds of death or dependency (modified Rankin Score mRS 3–6) at three to six months after a stroke (odds ratio (OR) 0.85, 95%CI 0.78–0.93) [10]). When administered within three hours of the onset of IS (11 trials, *n* = 2187), death or dependency was even more significantly reduced (OR 0.66, 95% CI 0.56–0.79) without increasing mortality. The early administration of intravenous thrombolysis (IVT) within 4.5 h of acute IS onset among patients without contraindications is now the standard of care [3]. Patients treated with IVT from 4.5 to 9 h after stroke onset, or wake-up stroke, would also benefit if they had imaging evidence of a perfusion mismatch [11].

Mechanical (also called endovascular) thrombectomy (EVT) involves the physical removal of clots in large intracranial arteries using a device passed intra-arterially to the level of the clot in a technique that uses cerebral angiography to visualize the vasculature. A meta-analysis of five large landmark trials of acute IS patients with occlusion of the proximal anterior circulation artery treated by EVT within 12 h of onset (*n* = 1287) showed that EVT significantly reduced disability as measured by the mRS at 90 days compared with a control (adjusted cOR 2.49, 95% CI 1.76–3.53) [12]. In a meta-analysis of nine randomized controlled trials combining IVT with EVT (*n* = 3740) versus EVT alone, the combination group was superior in functional independence (mRS 0–2) (OR 1.27, 95% CI 1.11–1.46) [13]. In a systematic review and meta-analysis of 16 studies (*n* = 7572) where intra-arterial thrombolysis was added to EVT, functional independence (mRS 0–2) at 90 days was non-significantly different (OR 1.14, 95%CI: 0.95–1.37) [14]. Thrombectomy within 24 h of onset among stroke patients with proximal large artery occlusion and a salvageable penumbra, with bridging IVT if it can be initiated within 4.5 h, is now the standard of care [3]. EVT has shown a functional benefit even among those with significant brain damage, as evidenced by an Alberta Stroke Program Early Computed Tomography Score of 3–5 or a calculated infarct volume of >50 mL [15].

## 3. Ultrasound

Sound is a form of mechanical energy [16]. Sound occurs when its source vibrates and produces cyclical oscillations of longitudinal waves that then allow the propagation of the energy. There are two phases in a sound wave—compression, which is the high-pressure phase of a sound wave, and rarefaction, which is the low-pressure phase. Sound needs a medium for it to travel—it is unable to travel in a vacuum. The frequency of an ultrasound is the number of wave cycles per second produced by the source—it is expressed in hertz (Hz). Sound waves conducted at a frequency above the upper limit of human hearing of 20 kHz (i.e., above 20,000 Hz) are called ultrasound. Ultrasound frequencies for clinical use range from 1 MHz to 20 MHz (i.e., 1 million to 20 million Hz).

When ultrasound enters tissue, a small amount is reflected by highly reflective objects back to the source/transducer, which forms the basis of diagnostic ultrasound imaging [16]. The sound waves may also be refracted if they pass through a different medium and thus shift direction. They may be scattered in multiple directions, or they may be absorbed, which forms the basis of therapeutic ultrasound. The absorbed energy causes vibration, heating, and the occurrence of vapor- or gas-filled cavitations/bubbles, as well as circulation of the liquid and the appearance of oxidation products [17].

Cavitation occurs when ultrasound passing through a liquid medium interacts with gaseous inclusions in that medium, e.g., microbubbles (MBs) [18]. Cavitation is broadly divided into stable cavitation (also called gas body activation (formerly identified as stable cavitation)), and inertial cavitation (also called transient cavitation). In stable cavitation, a relatively low level of ultrasound intensity activates a pre-existing gas body that resonates and undergoes periodic and regular volume changes in response to the applied acoustic pressure. Inertial cavitation requires a relatively higher ultrasound intensity that makes the MBs also undergo periodic volume changes in sync with the applied acoustic pressure, but the rapid increases in size lead to the bubble becoming unstable and then violently imploding.

## 4. In Vitro and In Vivo Effects of Ultrasound on Clots

The effect of ultrasound on blood clots has long been studied, with laboratory evidence in the 1950s showing that it is able to compress and retract clots [19,20]. While high-intensity ultrasound is able to mechanically fragment clots [21], low-intensity ultrasound is able to augment enzymatic fibrinolysis through non-thermal mechanisms by improving entry of fibrinolysis activators into the clot [22], reversibly altering fibrin structure and increasing tPA binding to fibrin [23]. The main mechanisms for clot lysis by ultrasound include acoustic stable cavitation and radiation force [24]. The use of ultrasound to cause or enhance clot lysis is called ‘sonothrombolysis’.

Among the earliest studies of therapeutic ultrasound for clot lysis, Sobbe et al. [25], building on earlier work by Ehringer et al. [26], showed that ultrasound was able to recanalize thrombosed femoral arteries in dogs without complications. Transcutaneous ultrasound applied in vivo combined with streptokinase was able to significantly augment the lysis of thrombi in the iliofemoral arteries of rabbits [27].

High-intensity focused ultrasound (1.5 MHz) initiated thrombolysis effectively and safely in a rabbit model of embolic stroke [28]. Ultrasound combined with a recombinant tissue plasminogen activator (rTPA) was more effective than rTPA treatment alone in reducing infarct volume in an embolic rat stroke model [29].

The value of magnetic resonance imaging (MRI)-guided focused ultrasound (MRgFUS) has been investigated. In a study using MRgFUS and monitoring using an MR angiogram (MRA) performed at 1-minute intervals, a 1 MHz ultrasound combined with rTPA was better able than rTPA alone to dissolve clots in the carotid artery of New Zealand rabbits [30].

Studies also evaluated the MRgFUS system through a plastic phantom skull. When combined with thrombolytic drugs and using transducers at either 0.5 or 1 MHz, clots could be destroyed in this in-vitro model [31].

In a study using a human temporal bone, a 1.8-MHz pulsed-wave (PW) ultrasound was passed through a human temporal bone onto whole blood clots. While clot weight was reduced using ultrasound alone compared to controls, an even greater reduction was achieved when ultrasound was combined with rTPA compared with rTPA alone [32].

To explore the impact of the human calvarium, artificial thrombi were placed inside the skull and located at the transducer focus point. Clot lysis increased with increasing acoustic output powers [33]. In another study, longer duty cycles combined with longer pulse widths had the highest potential to lyse clots [34].

Lower-frequency ultrasound in the kHz range, compared to the MHz range, is less attenuated by bone and is thus able to pass through the skull more easily [35]. Embolic rat models have shown that low-frequency ultrasound is able to significantly reduce infarct volume compared to pure TPA treatment. Animal studies did not show an increased rate of bleeding or harm to the blood–brain barrier.

There are reasonable concerns as to the formation of clot fragments when the clot is broken down by ultrasound, leading to the risk of distal embolization of these fragments and subsequent vascular occlusion. Rosenschein et al. showed in an animal model that 93% of clot fragments from sonothrombolysis were sub-capillary in size (<8 microns) [36] and thus unlikely to cause significant distal vascular occlusion. Other researchers have also reported very small fragments, 96% being <5 microns [37] or all being <11 microns [38]. It can be expected that the body’s endogenous fibrinolytic system would dissolve fragments, a process further enhanced by the co-administration of thrombolytic agents.

## 5. Microbubbles

Further progress came with the increased understanding of MBs. Under conditions of inertial cavitation, due to Bjerknes acoustic radiation forces and large-amplitude oscillations of MBs, there is a mechanical effect causing thrombus pitting and deformation [39]. The expansion and collapse of bubbles are able to stretch individual fibers within clots, while the dissipation of acoustic energy is able to break individual fibers in the clot—if the bubble located outside the clot collapses asymmetrically, an impinging jet is created that aids in clot destruction [40].

MB-mediated sonothrombolysis is a complex process involving the clot, ultrasound, thrombolytic drug, and MBs. Clot lysis is best achieved by combining ultrasound at a high acoustic pressure with TPA and MB, compared to a single agent or in any other combination [41]. A similar result was seen an in vitro circulating flow model evaluating the role of tenecteplase (TNK-tPA)-mediated thrombolysis of fully retracted porcine blood clots where higher FUS frequencies (1 MHz) are associated with better thrombolysis compared to lower FUS frequencies (0.6 MHz), and an even better thrombolytic efficacy of the combination of 1-MHz FUS pulses with TNK-tPA and MBs [42]. In a thromboembolic stroke model of middle cerebral artery occlusion (MCAO) in rats, MB-mediated sonothrombolysis led to a similar reduction in infarct volume compared to rTPA [43]. A systematic review of 16 pre-clinical studies found heterogeneity in ultrasound parameters and types of MBs used, but four studies showed the superiority of sonothrombolysis over rTPA based on clinical criteria; however, safety data was limited [44].

Contrast agents are MBs that are given intravenously, are sufficiently small and stable enough to survive the cardiopulmonary circulation, and remain in the circulation to then enter other vascular beds, e.g., the cerebral circulation [45]. These MBs increase the Doppler signal by 10 dB to 30 dB, allowing the detection of flow in locations for which attenuation may make successful insonation impossible, e.g., the transcranial examination of intracranial vessels, especially through unfavorable bone windows. They can increase the success rate of the examination, reduce the time required for the examination, and facilitate the detection of a greater number of vessels. 

Contrast agents and MB formulations used in animal models include Sonovue, Definity, perflutren lipoid, albumin, and BR38 [46,47,48,49,50]. Magnetic targeting of MBs using a single magnet allows for increased lysis rates [51].

New research has shown that different shapes showed different cavitation reactivity, e.g., of two different gold nanoparticles in shape functionalized with the rtPA, rtPA-functionalized asymmetric gold nanostars (NSt) were superior to gold nanospheres (NPt) in causing cavitation [52]. As clots fragmented by sonothrombolysis may result in debris that then migrates distally and causes further occlusions, phase-change nanodroplets can reduce the overall clot debris size and may allow for safer sonothrombolysis [53].

More recent techniques involve the intraluminal visualization of clots and the delivery of ultrasound intravascularly [54]. Intra-arterial B-mode ultrasound imaging was able to verify intra-clot delivery and clot penetration of the concurrently administered ultrasound contrast agents Optison and SonoVue with rTPA [55]. MBs may also be delivered intra-arterially while the ultrasound is applied from an extracranial source [56]. A dual-mode ultrasound catheter that combined a 16-MHz high-frequency element (imaging transducer) and a 220-kHz low-frequency element (treatment transducer) showed successful in-vitro sonothrombolysis [57].

Hollow nanogels loaded with thrombolytic agents, e.g., urokinase-type plasminogen activator (uPA), octafluoropropane, and rTPA-loaded echogenic liposomes (OFP t-ELIP), when sonolysed at the site of the clot, allow the delivery of thrombolytic agents at the site of the thrombus [58].

In parallel, there has been a greater understanding of how the characteristics of a clot may affect its susceptibility to sonothrombolysis. Sonothrombolysis is more successful on fresh thrombi and those with high cholesterol levels within it [59]. Platelet-rich clots are resistant to thrombolysis, which may explain some of the failures in recanalization [60]. Higher acoustic pressure is needed to lyse arterial thrombi compared to venous thrombi [61].

## 6. Transcranial Ultrasound Imaging

The intracranial structures can be imaged in various ways, including by computed tomography (CT) and MRI [62]. The vasculature can be studied by CT angiography (CTA), where intravenous contrast is injected and high-resolution small-thickness slice images are rapidly taken of the brain and blood flow through the vessels and usually reconstructed into easily interpretable images—while a rapid technique and widely available, it does carry a radiation risk and needs a contrast injection which can be an issue in those with renal dysfunction or a contrast allergy. MR angiography (MRA) is another method where the signals generated by flowing blood in a magnetic field are used to again reconstruct easily interpretable images of blood flowing through the vessels—while it carries no radiation risk and does not usually require contrast injection, it may not be easily available especially after office hours if at all, takes much longer than CTA, and may be a challenge in claustrophobic or clinically unstable patients, or those with pacemakers.

Ultrasound imaging has the advantages of being safe, portable, and rapid, and is usually widely available—transcranial Doppler (TCD) is routinely performed on stroke centers to evaluate blood flow in the basal cerebral arteries in acute stroke [63]. Transcranial color-coded duplex (TCCD) imaging marries the blind TCD technique with B-mode imaging and color coding of Doppler signals generated by flowing to allow a more accurate ultrasound visualization of the intracranial vessels and is also used in stroke centers as a complementary technique to study blood flow [64]. Both TCD and TCCD involve passing low-frequency ultrasound waves in the 2 MHz range through the intact skull to visualize flowing blood and can diagnose arterial stenosis, vasospasm, occlusion, compensatory flow, and embolization, to name a few [65].

## 7. Clinical Case Series

There have been a few publications reporting the clinical experience with sonothrombolysis for acute ischemic stroke (Table 1).

Among the earliest case series was by Alexandrov et al. in 2000, where 40 patients with acute IS of a moderate severity were monitored using a 2 MHz TCD while they were receiving rTPA [66]. They found recanalization in 70% of patients, with dramatic recovery during rTPA infusion in 20%, all of whom had full recanalization. At 24 h, 40% had significantly improved or recovered fully; despite a small risk of hemorrhage. The authors felt that ultrasound may result in the exposure of a greater amount of the clot surface and thus assist in thrombolysis.

Another publication in 2002 by Cintas et al. using TCCD in six severe IS patients with MCA occlusion but no thrombolytic agent showed at least partial recanalization in 83%, with a reduction in the neurological deficit over the first 24 h; they did not find any hemorrhage [67].

Alexandrov et al. built on their previous work by performing a combined lysis of thrombus in brain ischemia using transcranial ultrasound and systemic TPA (CLOTBUST) trial [68]. Their Phase 1 data on 55 moderately severe IS patients with arterial occlusion receiving rTPA and TCD, again documented complete recanalization in 36% by 2 h and dramatic or at least significant improvement at 24 h in 49%, but a small risk of hemorrhage.

Brunser et al. treated 61 moderately severe acute IS patients, most with MCA occlusion, attending their clinic with thrombolysis and TCD [69]. Complete recanalization occurred in 44.3%; at three months, 60% attained a modified Rankin score (mRS) of 0–2, with a 10% case fatality. Gu et al. reported their experience with 20 IS patients treated with thrombolysis and TCD, which resulted in full or partial recanalization in 36%, with 76% being ADL-independent at three months [70]. Aaron et al. reported on their 18 IS patients receiving thrombolysis and TCD, with immediate dramatic improvement in 30%, which reached 50% in the next 24 h [71]. Despite the small hemorrhagic risk and 11% mortality at six months, 63% attained an mRS ≤ 2.

## 8. Case-Controlled Studies and Non-Randomized Clinical Trials

While case series are able to provide useful initial information, comparing with suitable controls would allow a more accurate understanding of outcomes (Table 2).

Encouraged by animal data on the safety and efficacy of low-frequency ultrasound [35], Daffertshofer et al. performed a non-randomized transcranial low-frequency ultrasound-mediated thrombolysis in brain ischemia (TRUMBI) clinical trial using 300 kHz ultrasound among moderately severe acute IS patients with documented vascular occlusion already receiving rTPA [72]. The study was stopped prematurely due to significantly higher bleeding rates in those receiving ultrasound. Subsequent simulations showed that low-frequency ultrasound led to large standing waves and peak rarefactional pressure that was higher than the inertial acoustic cavitation threshold in large areas of the brain, even away from the targeted clot, leading to hemorrhage [73].

**Table 2:** Case-controlled studies and non-randomized trials of sonothrombolysis for acute ischemic stroke.

Author	Daffertshofer M, et al. [72]	Perren F, et al. [74]	Rubiera M, et al. [75]	Dinia L, et al. [76]	Bardon P, et al. [77]	Dwedar AZ, et al. [78]	Reinhard M, et al. [79]
Year of publication	2005	2008	2008	2009	2012	2014	2015
Number of patients	26 (14 vs. 12)	26 (11 vs. 15)	138 (91 vs. 47)	236 (138 vs. 98 historic controls)	49 (12 bilateral TCD vs. 37 unilateral TCD)	42 (21 vs. 21)	132 (73 EVT vs. 59 ST)
Age (yr)	70.4 ± 9.7	61 ± 27			Average 64.1 ± 9.4 Range 47–78 vs. Average 62.2 ± 12.1 Range 32–78	Mean 59.2 ± 10.6 vs. 59.2 ± 9.3 Range 40–70	Median 71 (IQR 60–78) vs. 75 (IQR 61–82)
Time window	Mean 2.42 ± 0.50 h (range 0.50–4.35 h)	≤3 h	178 min (mean)	≤3 h	Mean 133.8 ± 58.4 min vs. 142.3 ± 56.6 min	Mean 5.02 ± 1.2 h vs. 8.0 ± 2.2 h	Median 117 min (IQR 95–116) vs. 105 (IQR 83–148) and 234 (IQR 187–325) til DSA
Stroke type	‘Vascular obstruction’		MCA occlusion		MCA occlusion	MCA occlusion	M1 or carotid T occlusion
Technique	TCD	TCCD	TCD	TCD	TCD	TCD	TCD
Frequency	300 kHz	2 MHz	2 MHz	2 MHz	2 MHz	2 MHz	1.6 MHz
Concomitant thrombolysis	Yes	Yes, plus Sonovue contrast agent perfusion	Yes, plus Levovist (galactose-based air-filled MB)	Yes, plus 3 doses of 2.5 g of MB after tPA bolus	No vs. yes	No	Yes
Comparator	No USG	No Sonovue	Sonovue (sulfur hexafluoride-filled MB) instead of Levovist	Historic controls	60-min bilateral 2-MHz pulsed-wave Doppler monitoring of the area of occlusion vs. standard sonothrombolysis	Patients who did not receive 1 h continuous TCD	Thrombectomy
Outcome	Partial or complete recanalization—28.6 vs. 50% (*p* = 0.2629)	Recanalization 64% vs. 53% Patients who received ECA improved their NIHSS significantly more than those who were only TCCD monitored (Mann–Whitney U = 48.0; *p* = 0.050), and their flow signal improved more (Mann–Whitney U = 40.0; *p* < 0.03).	Recanalization rates after 1 h (32.2% vs. 35.6%), 2 h (50.0% vs. 46.7%) and 6 h (63.8% vs. 54.5%) (*p* > 0.3). Clinical improvement (NIHSS decrease ≥ 4 points) at 24 h (54.9% vs. 51.1%), (*p* = 0.400) mRS ≤ 2 at 3 mo—44% vs. 48.5%	Recanalization rates higher in the MB compared with the control group at 1, 2, 6, and 12 h (*p* < 0.05). MB administration associated with higher degree of clinical improvement at 24 h (54.9% vs. 31.1%, *p* = 0.004)	Complete recanalization found in 30.0% of Group 1 and 32.4% of Group 2 Independent at 90 days—58.3% in Group 1 vs. 59.5% in Group 2	Mean flow velocity (MFV) in MCA one after the initial study, at 20 and 60 min—change in MFV after insonation for Group 1 in comparison to Group 2 at 3 time points was significantly high (*p* < 0.001).	Functional independence (mRS 0–2) higher for EVT (adjusted OR 3.89 (95% CI 1.36–12.58)) Ordinal mRS analysis favored EVT (adjusted common OR 1.70 (95% CI 0.88–3.31)).
Adverse events	Bleeding in MRI—92.9% vs. 33.3% (*p* < 0.01)	Symptomatic intracranial hemorrhage 9% vs. 7%	Symptomatic intracranial hemorrhage rate (3.3% vs. 2.1%, *p* = 0.580)	MB administration associated with an increased risk of hemorrhagic infarction (HI1-HI2 (21% vs. 12%, *p* = 0.026) Parenchymal hematoma (PH1-PH2 and symptomatic intracranial hemorrhage)—similar in both groups	Symptomatic intracranial hemorrhage—2.7% of the Group 2	Nil	Symptomatic intracerebral hemorrhage—4.1% In EVT group

In a non-randomized clinical trial using TCCD, Perren et al. investigated the added value of adding the contrast agent Sonuvue to thrombolysis and ultrasound in 11 patients versus 15 controls. Those receiving contrast had improved flow signals and NIHSS scores [74].

Rubiera et al. compared the effects of two different MBs—galactose-based air-filled MBs (Levovist) (*n* = 91) and sulfur hexafluoride-filled MBs (Sonovue) (*n* = 47)—on recanalization and clinical outcomes among patients already receiving thrombolysis [75]. Recanalization rates, clinical improvement functional outcome, and symptomatic intracranial hemorrhage (ICH) rates were similar between the two groups.

Dinia et al. studied the risk of hemorrhagic transformation of administering MBs after thrombolysis and TCD for 138 IS patients, compared to 98 historical controls [76]. While the MB group had better recanalization and clinical improvement, there was also an increased risk of hemorrhagic transformation, but not symptomatic intracranial hemorrhage.

Bardon et al. conducted a pilot study comparing bilateral TCD monitoring among 12 IS patients contraindicated for thrombolysis, compared with 37 patients receiving thrombolysis and unilateral TCD—they found no difference in recanalization rates or 90-day functional outcomes [77].

In a study by Dwedar of moderately severe IS patients with MCA occlusion not receiving thrombolysis in 21 cases receiving TCD and 21 not receiving TCD, there were significantly better blood flow velocities among the cases with no bleeding events [78].

In this era of MT, Reinhard et al. compared EVT (*n* = 73) versus sonothrombolysis (*n* = 59) in acute IS patients with proximal MCA or carotid-T occlusion [79]. While they found that EVT led to better functional independence at the end of neurorehabilitation, this may apply more to the carotid-T occlusion group. EVT did carry a small bleeding risk.

## 9. Randomized Controlled Trials

Adequately powered randomized controlled trials (RCTs) are considered the gold standard to assess the efficacy and safety of medical interventions. There have been a number of RCTs of sonothrombolysis for acute stroke published since 2003 [80,81,82,83,84,85,86,87,88] (Table 3).

Eggers et al. were among the first to report a controlled trial involving patients with acute MCA occlusion who were receiving rTPA, with 11 undergoing continuous TCCD and the 14 controls not undergoing TCCD [75]. While there was a higher hemorrhagic rate among the cases, they also had a higher recanalization rate and a better functional outcome. The authors had similar findings in a similar trial conducted five years later [85].

The CLOTBUST trial by Alexandrov et al. built on their earlier work [66,68] by equally randomizing 126 patients with acute ischemic stroke from MCA occlusion receiving intravenous t-PA within three hours after the onset of symptoms to either receive continuous 2-MHz transcranial Doppler ultrasonography or a placebo [81]. The primary combined end point of complete recanalization as assessed by transcranial Doppler ultrasonography or dramatic clinical recovery at 24 h was seen significantly more in the intervention arm, but dramatic clinical recovery at 24 h and good functional outcome (mRS 0–1) at three months was not different.

As a corollary to their earlier trial [80], Eggers et al. studied the effect of TCCD on 15 patients with acute MCA occlusion who were contraindicated for thrombolysis [82]. By day four, recanalization and neurologic improvement were more frequent in the intervention group. There was no increase in hemorrhage. Ultrasound may have a role in clot lysis for patients contraindicated for thrombolytic therapy.

Molina et al. continued their work on MBs [83] by performing a three-arm trial comparing rTPA alone (*n* = 36), rTPA plus TCD (*n* = 37), and both with three doses of MBs (*n* = 38) among 111 patients with MCA occlusion [78]. Triple therapy led to the best recanalization and neurological outcome, with an increase in the risk of hemorrhage.

Alexandrov et al. performed a pilot safety trial of adding perflutren-lipid microspheres to TCD with thrombolysis in four cases: one control ratio in 15 patients with MCA occlusion [84]. There was no increase in hemorrhagic risk. Recanalization was better with the microspheres group than in the control arm of CLOTBUST [81].

MRX-801 microspheres were investigated by Molina et al. in the TUSCON trial of low vs. high vs. no microspheres among 36 patients with proximal intracranial occlusion receiving rTPA and TCD [86]. While recanalization was better among those receiving microspheres, the higher dose was associated with increased hemorrhage.

The NOR-SAS clinical trial by Nacu et al. involved administering the ultrasound contrast agent Sonuvue to 183 acute ischemic stroke patients also receiving TCD and rTPA [87]. There was no impact on neurological or functional outcome, or hemorrhage.

The most recent trial was CLOTBUST-ER by Alexandrov et al., delivering ultrasound using an operator-independent device to 676 patients with acute ischemic stroke receiving rTPA [88]. While safe, there was no improvement in functional outcome.

Most trials had small numbers except the one by Alexandrov et al. in 2019 [88]; most had time windows < 3 h; most used TCD, although Eggers et al. used TCCD in all their trials [80,82,85]; the ultrasound frequency was 1.8 to 2 MHz; almost all had concomitant rTPA except for the one by Eggers in 2005 [82]; and two by Molina et al. [83,86] and one by Alexandrov et al. [84] also tested the addition of MBs or microspheres [84,88].

Chen et al. performed a meta-analysis of seven RCTs (*n* = 549) of sonothrombolysis versus non-sonothrombolysis for acute IS [89]. Compared to no sonothrombolysis, sonothrombolysis improved complete recanalization (RR 2.16, 95% CI 1.51 to 3.08, *p* < 0.001), complete or partial recanalization (RR 1.90, 95% CI 1.26 to 2.88, *p* = 0.002), with a tendency to improvement of ≥4 points in NIHSS score (RR 1.43, 95% CI 0.99 to 2.07, *p* = 0.057). However, there were no significant differences in neurological recovery and adverse events. In a subgroup analysis, factors that may improve efficacy outcomes included concomitant use of t-PA, severe stroke (NIHSS > 15), treatment time ≤ 150 min, and a relatively younger age (≤65 years).

However, this meta-analysis did not include the largest trial by Alexandrov et al. [88]. A subsequent individual patient data meta-analysis of patients with proven large vessel occlusion did include this trial [90]. Among the seven randomized controlled clinical trials (*n* = 1102), 138 were randomized to treatment and 134 to control. Patients receiving sonothrombolysis were more likely to achieve complete recanalization compared with those receiving IVT only (40.3% vs. 22.4%; OR 2.17 [95% CI, 1.03–4.54]; adjusted OR, 2.33 [95% CI, 1.02–5.34]). There was no significantly increased risk of symptomatic intracranial hemorrhage (7.3% versus 3.7%; OR, 2.03 [95% CI, 0.68–6.11]; adjusted OR, 2.55 [95% CI, 0.76–8.52]). The likelihood of asymptomatic intracranial hemorrhage and functional independence at three months was similar.

A recent analysis [91] of studies using MBs and ultrasound in peripheral arterial thrombosis reviewed eight clinical stroke studies, including two case series [92,93]. There was high heterogeneity among the studies, which made it impossible to perform a meta-analysis. Still, overall, recanalization rates were higher in the groups that underwent contrast-enhanced sonothombolysis.

## 10. Progress in Ultrasound Delivery to Patients

The traditional ultrasound beam at a frequency of 2 MHz allows for the insonation of deeper structures but suffers from severe attenuation by the skull and the highly demanding skill needed to focus the beam at the site of occlusion [63]. The initial promise of an easy-to-use device using a low frequency of 300 kHz that would transgress the skull easily and not need precise targeting of the occlusion [33] was broken when the TRUMBI trial showed an unacceptable risk of ICH [72]. Progress was made when a hands-free, operator-independent device was developed and shown to be safe before being tested in the CLOTBUST-ER clinical trial [88].

Delivery of ultrasound intra-arterially was tested using the EkoSonic Endovascular System in a prospective study of 14 patients, mean age 65.1 ± 11.2 years, with moderate acute IS and occluded middle cerebral artery (*n* = 7) or basilar artery (*n* = 7), compared with historical controls [94]. Endovascular sonolysis was started within 8 h of stroke onset. Arterial recanalization was achieved in 85.7% of patients with MCA occlusion and 100% with BA occlusion. There were no symptomatic intracerebral hemorrhage nor periprocedural complications. The median mRS at 90 days was one (IQR 1–3.5) in MCA occlusion and three (IQR 2–4.5) in BA occlusion, compared to five (IQR 305) and six (IQG4.5–6), respectively, among the controls.

## 11. Stroke Prevention

In view of the risk of new brain ischemic lesions that may occur in up to two-thirds of patients undergoing coronary artery bypass grafting or cardiac valve surgery, Skoloudik et al. found in the randomized controlled SONORESCUE trial (*n* = 60 vs. 60, mean age 65.3 yr) that intra-operative sonolysis would reduce the risk of new lesions > 0.5 mL as well as the median volume of new ischemic lesions on diffusion-weighted imaging (DWI) MRI in this group of patients [95]. Clinical stroke or transient ischemic attack (TIA) occurred in 0% of treated vs. 3.3% of untreated patients (*p* = 0.496). The subsequent SONOREDUCE randomized controlled trial involving 144 patients undergoing coronary stenting only was neutral [96].

As silent brain infarctions may occur in up to one-third of patients undergoing carotid endarterectomy and two-thirds of those undergoing carotid angioplasty and/or stenting, Skoloudik et al. investigated in the randomized controlled SONOBUSTER trial the use of intraoperative sonolysis in these patients [97]. They found that new lesions on an MRI were again less frequent among the 121 patients (mean age, 66.65 ± 7.17 years) compared to 121 controls (75; 66.02 ± 8.11 years). Clinical stroke or TIA occurred in 0.8% vs. 2.5% respectively (*p* > 0.3).

In the randomized controlled SONOBIRDIE trial of sonolysis of patients while they were undergoing carotid endarterectomy, (*n* = 1004, mean age 68 ± 7.8 years), the primary composite outcome of ischemic stroke, TIA, or death within 30 days occurred in 2.2% vs. 7.6% in controls (risk difference 5.5%; 95% CI 2.8–8.3%; *p* < 0.001)—stroke/TIA occurred in 1.8% vs. 7.5% (*p* < 0.001) while a new ischemic lesion on an MRI was seen in 8.6% vs. 17.4% of patients (*p* < 0.01) [98].

## 12. Conclusions

Emergent recanalization of occluded arteries remains the holy grail of hyperacute therapy in ischemic stroke. IVT and, more recently, EVT for carefully selected patients are now the standard of care. Transcranial ultrasound either as TCD or TCCD, alone or in addition to IVT, for occluded arteries, in the 2 MHz range, has been shown in vitro and in vivo, both in animal models and humans, to aid thrombolysis and improve recanalization without significantly increasing the risk of symptomatic intracranial hemorrhage. The use of 300 kHz ultrasound is unsafe. The adjunctive use of MBs or microspheres has not shown a benefit in randomized controlled trials. Further evidence is needed on the clinical value of endovascular ultrasound. Other techniques explored in animal models, including the endovascular delivery of MBs, the use of IVTMBs filled with thrombolytics, and differentially shaped nanoparticles, await clinical trials. While much has been done, much more lies ahead as evidence is generated for the clinical role of sonothrombolysis in the management of acute ischemic stroke.

## Figures and Tables

**Table 1:** Case series of sonothrombolysis for acute ischemic stroke.

Author	Alexandrov AV, et al. [66]	Cintas, et al. [67]	Alexandrov AV, et al. [68]	Brunser A, et al. [69]	Gu T, et al. [70]	Aaron S, et al. [71]
Year of publication	2000	2002	2004	2014	2015	2017
Number of patients	40	6	55	61	20	14
Age (yr)	Mean 70 ± 16	Mean 54.3 ± 14.7	Mean 69 ± 15	Mean 66 ± 18	Median 71 (IQR 63–82)	Mean 55 (range 32–76)
Time window (min)	Mean 125 ± 52	Mean 210 ± 86	Mean 125 ± 36	Mean 127	Median 98 (IQR 67–131)	Mean 138 (Range 65–256)
Stroke type	‘Occluded vessels’ (MCA. ICA, BA)	MCA occlusion	‘Proximal arterial occlusion’	MCA occlusion	‘Flow obstruction’	LA-AIS
Technique	TCD	TCCD	TCD	TCD	TCD	TCD
Frequency (MHz)	2	2	2	2	2	2
Concomitant thrombolysis	Yes	No	Yes	Yes	Yes	Yes
Comparator	Nil	Nil	Nil	Nil	Nil	Nil
Outcome	Recanalization found at 45 ± 20 min—complete in 30%, partial in 40%. Dramatic recovery (total NIHSS score < 3)—20%)—all had complete recanalization. Improvement by ≥10 NIHSS points or complete recovery—30% at the end of tPA infusion, 40% at 24 h Improvement by ≥4 NIHSS points—62.5% at 24 h 3-month mortality—20%	Partial recanalization (blunted waveforms)—83%. Mean time to beginning of recanalization 17.2 ± 9.6 min. Complete recanalization at 24 h—16.6% Mean NIHSS score in patients who recanalized during monitoring—21.2 ± 4.1 at baseline, 19.2 ± 5 at 2 h, and 15.6 ± 3.4 at 24 h	Complete recanalization within 2 h 36%. Dramatic recovery (NIHSS score ≤ 3)—20% at 2 h, 24% at 24 h. Improvement by ≥4 NIHSS points—49% at 24 h	Complete recanalization—44.3%. Modified Rankin Scale of 0–2 at 3 months—60% Case fatality—9.8%	Full recanalization—29% Full or partial recanalization—36% 3-month follow-up—76% ADL independent	TIBI residual flow grade of ≥2—83%. Immediate dramatic improvement (NIHSS score ≤ 3 points or improvement by ≥10 points)—30%; within the next 24 h—50%. At 6 months—mortality 11%, mRS ≤ 2 63%
Adverse events	Symptomatic intracerebral hemorrhage—7.5%	No hemorrhage	Symptomatic hemorrhage—5.5%	Asymptomatic intracranial hemorrhage—9.8%	No symptomatic intracranial bleeding	Symptomatic hemorrhage—5.5%

**Table 3:** Randomized controlled trials of sonothrombolysis for acute ischemic stroke.

Author	Eggers J, et al. [80]	Alexandrov AV, et al. [81]	Eggers J., et al. [82]	Molina CA, et al. [83]	Alexandrov AV, et al. [84]	Eggers J, et al. [85]	Molina CA, et al. [86]	Nacu A, et al. [87]	Alexandrov AV, et al. [88]
Year of publication	2003	2004	2005	2006	2008	2008	2009	2017	2019
Number of patients	11 vs. 14 controls	63 vs. 63 controls	8 vs. 7	38 vs. 37 vs. 36	12 vs. 3	19 vs. 18	12 vs. 11 vs. 12	93 vs. 90	335 vs. 341
Age (yr)	61 ± 9	67 ± 12 70 ± 13	58.9	68 ± 12 vs. 70 ± 9 vs. 68 ± 11	75 ± 13 vs. 58 ± 33	61 ± 10		68.8 ± 16.2	70
Time window	144.2 min	150 vs. 130 min	213.4 min	158 ± 35 vs. 161 ± 38 vs. 152 ± 33	<3 h	143.2	<3 h	170 ± 69	121.5
Stroke type	MCA occlusion	MCA occlusion	MCA occlusion	MCA occlusion	MCA occlusion	MCA occlusion	‘Proximal intracranial occlusion’	Acute IS	Acute IS
Technique	TCCD	TCD	TCCD	TCD	TCD	TCCD	TCD	TCD	TCD
Frequency	2 MHz	2 MHz	2 MHz	2 MHz	2 MHz	1.8 MHz	2 Mz	2 MHz	2 MHz
Concomitant thrombolysis	Yes	Yes	No	Yes, plus TCD plus 3 doses of 2.5 g of MB after tPA bolus	Yes, plus 2.8 mL microS (perflutren-lipid microspheres)	Yes	Yes, plus microS (MRX-801) infusion 1.4 mL or 2.8 mL	Yes, plus TCD plus contrast	Yes
Comparator	No ultrasonography	No TCD	No USG	rTPA plus TCD vs. rTPA only	No microS	No USG	No microS (MRX-801)	No contrast	No USG
Outcome	Recanalization 27.3 vs. 21.4% 3-month favorable functional outcome 54.5% vs. 36.3%	Complete recanalization or dramatic clinical recovery within two hours after the administration of a t-PA bolus—49% vs. 30% *p* = 0.03). At 24 h, dramatic clinical recovery—44% vs. 40% (*p* = 0.7). At three months, mRS 0–1 42 % vs. 29% (*p* = 0.20).	Recanalization 62.5% vs. 0% 3-month favorable functional outcome 25% vs. 0%	Two-hour recanalization was seen in 71% vs. 68% vs. 39% >4 point improvement of NIHSS score at 24 h—55% vs. 41% vs. 31%	At 2 h, sustained complete recanalization—42% in treatment arm NIHSS scores 0 to 3—17% in treatment arm	Recanalization (complete or partial) after 1 h 57.9% vs. 22.2% (*p* = 0.045) US group showed greater improvement in National Institutes of Health Stroke Scale values at days 1 and 4 After 90 days, modified Rankin Score ≤1—21.1% vs. 0% (*p* = 0.106) Barthel Index ≥ 95—42.1% vs. 0% (*p* = 0.003)	Sustained complete 67% vs. 46% vs. 33% (*p* = 0.255) 3-month clinical recovery rates 75% vs. 50% vs. 36% (*p* = 0.167).	Neurological improvement at 24 h—51% vs. 46% 90 days mRS 0–1—48% vs. 51% Death 6% vs. 9%	Improvement in modified Rankin Scale score at 90 days—31.3% vs. 32.0% (*p* = 0.74). Mortality 16% vs. 13% (*p* = 0.37)
Adverse events	Symptomatic intracranial hemorrhage 18.2% vs. 0%	Symptomatic intracerebral hemorrhage 4.8% in both groups	Symptomatic intracranial hemorrhage 0% vs. 14.3%	Symptomatic intracranial hemorrhage 2.6% vs. 2.7% vs. 5.5%	Symptomatic intracranial hemorrhage 0% Asymptomatic intracranial hemorrhage 25% vs. 33.3%	Symptomatic intracranial hemorrhage 15.8% vs. 5.6%	Symptomatic intracranial hemorrhage 0% vs. 25% vs. 0%	Symptomatic intracerebral hemorrhage 2% vs. 4%	Symptomatic intracranial hemorrhage 2.5% vs. 1.5%

## Data Availability

No new data were created or analyzed in this study.
